# Supplementary material for: Gene Networks and Pathways Involved in LPS-Induced Proliferative Response of Bovine Endometrial Epithelial Cells
Source: Genes (Basel). 2022 Dec 12;13(12):2342. doi: 10.3390/genes13122342 (PMC9778113; doi:10.3390/genes13122342)
Supplement: Supplementary file 1 [file genes-13-02342-s001.zip › genes-2028257-supplementary.pdf]

Supplementary Materials

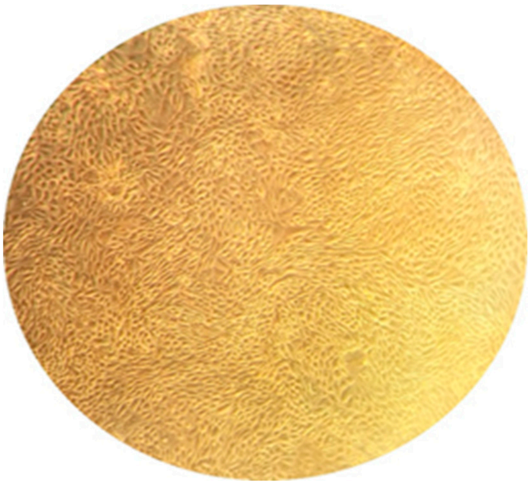

bEEC (98% purity)

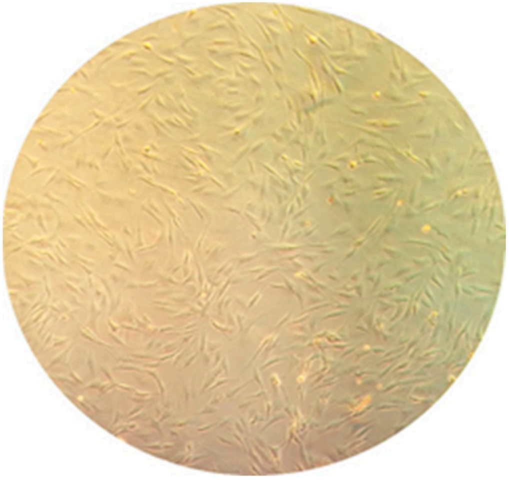

Bovine Fibroblst

A.

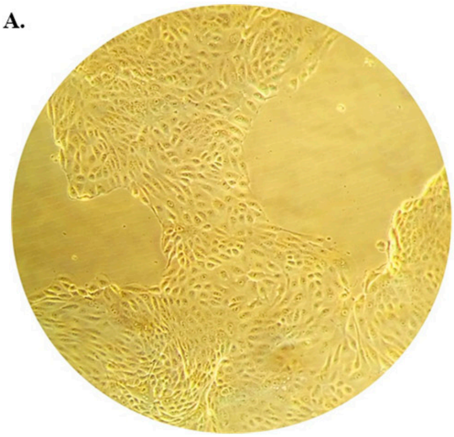

B.

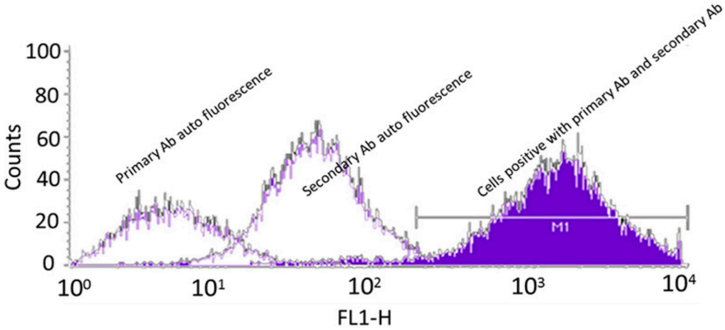

Figure S1: Morphology and purity of bovine fibroblast and endometrial epithelial cells. A. Cell morphology of bovine endometrial epithelial cells (bEECs) B. Representative Flow cytometric histogram of bEECs expressing the epithelial-specific cytokeratin-18 showing their appearance to the epithelial cell population. (98% of cells are in M1 region corresponding to this population) [1]. The change in cellular biology and physiology of bEECs following LPS were shown in previous work [17].

Table S1: Summary of RNA quality and RNA-seq reads mapping to reference genome [1].

Samples Cow	RNA (RIN)	Concentration	LPS (µg/ml)	Total reads	TrimmedReads. Both Surviving	MappedReads. Unique	% Unique mapped reads
		(ng/µl)		(× 10^6)	(× 10^6)	(× 10^6)	
No.1	10	1361	T0 *	28.49	28.43	26.89	94.58
	10	1037	C0 **	27.58	27.52	25.95	94.29
	9.9	805	2	28.16	28.10	26.53	94.41
	9.9	946	8	29.82	29.75	28.03	94.22
No.2	9.1	1018	T0 *	29.99	29.92	27.56	92.11
	8.8	1081	C0 **	30.08	29.98	26.82	89.46
	9.0	1450	2	29.19	29.12	26.39	90.63
	8.9	1417	8	28.78	28.71	26.75	93.17
No.3	10	1580	T0 *	26.11	26.05	24.58	94.36
	9.9	1227	C0 **	25.30	25.23	23.81	94.37
	9.8	1405	8	24.97	24.89	23.28	93.53
	9.8	1096	8	31.54	31.46	29.63	94.18
Average				28.33 ± 2.03	28.26 ± 2.03	26.35 ± 1.96	
Overall				340.01	339.16	316.21	

(T0 * = Time 0 control. no LPS; C0 ** = 24h control no LPS

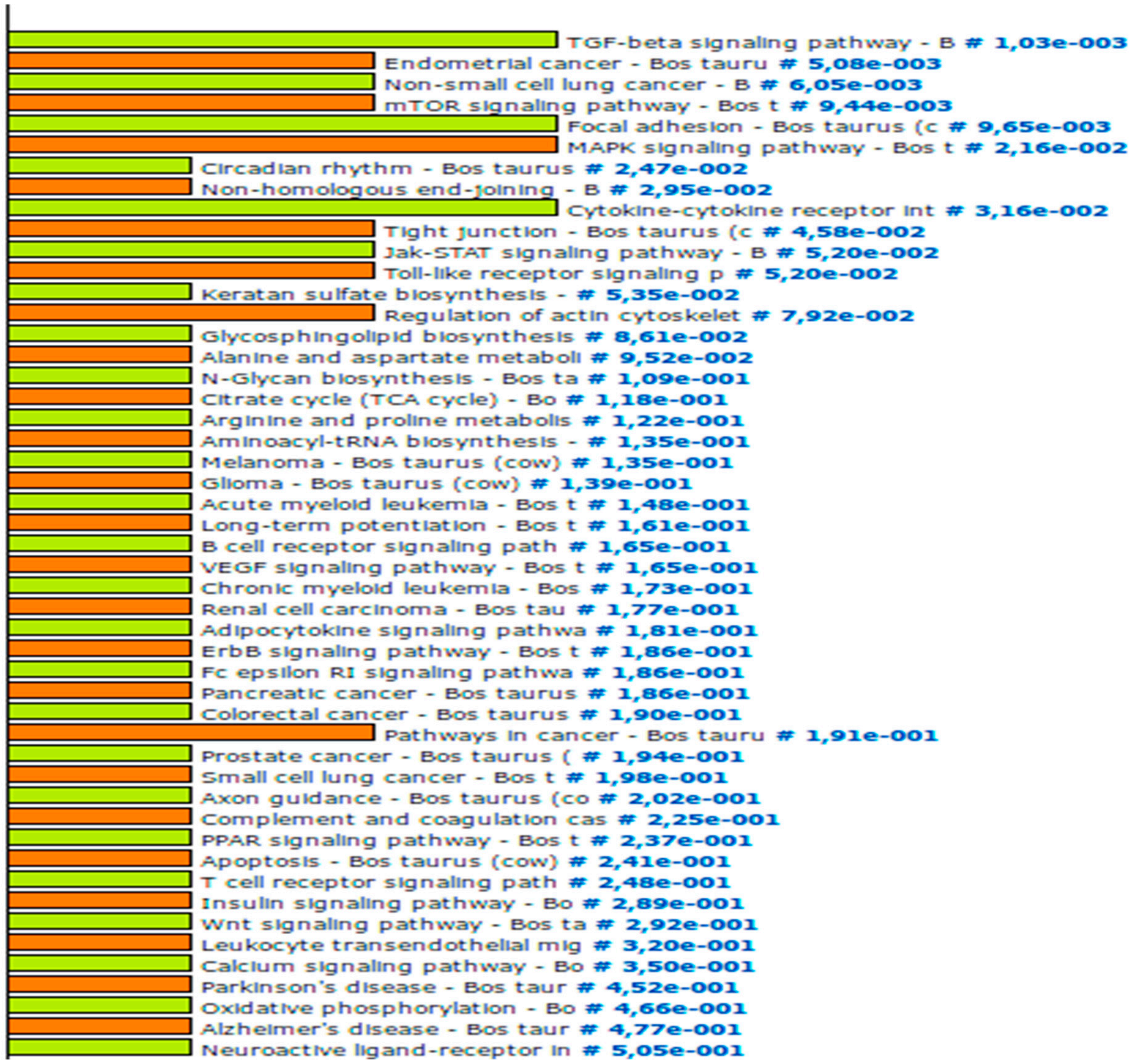

Figure S2: Biological pathways analysis of all DEGs in Gene browser platform. The graph displays the term name and the p-value (blue numbers). The length of the bars shows the number of genes that are involved in each pathway.
